# Investigations of In_2_O_3_ Added SiC Semiconductive Thin Films and Manufacture of a Heterojunction Diode

**DOI:** 10.3390/nano14100881

**Published:** 2024-05-19

**Authors:** Chia-Te Liao, Chia-Yang Kao, Zhi-Ting Su, Yu-Shan Lin, Yi-Wen Wang, Cheng-Fu Yang

**Affiliations:** 1 Department of Aviation Communication and Electronics, Air Force Institute of Technology, Kaohsiung 820, Taiwan; 2 Department of Chemical and Materials Engineering, National University of Kaohsiung, Kaohsiung 811, Taiwan; 3 Department of Aeronautical Engineering, Chaoyang University of Technology, Taichung 413, Taiwan

**Keywords:** In-doped SiC thin films, n-type semiconductor, X-ray photoelectron spectroscopy, heterojunction p-n junction, diode

## Abstract

This study involved direct doping of In_2_O_3_ into silicon carbide (SiC) powder, resulting in 8.0 at% In-doped SiC powder. Subsequently, heating at 500 °C was performed to form a target, followed by the utilization of electron beam (e-beam) technology to deposit the In-doped SiC thin films with the thickness of approximately 189.8 nm. The first breakthrough of this research was the successful deposition of using e-beam technology. The second breakthrough involved utilizing various tools to analyze the physical and electrical properties of In-doped SiC thin films. Hall effect measurement was used to measure the resistivity, mobility, and carrier concentration and confirm its n-type semiconductor nature. The uniform dispersion of In ions in SiC was as confirmed by electron microscopy energy-dispersive spectroscopy and secondary ion mass spectrometry analyses. The Tauc Plot method was employed to determine the Eg values of pure SiC and In-doped SiC thin films. Semiconductor parameter analyzer was used to measure the conductivity and the I-V characteristics of devices in In-doped SiC thin films. Furthermore, the third finding demonstrated that In_2_O_3_-doped SiC thin films exhibited remarkable current density. X-ray photoelectron spectroscopy and Gaussian-resolved spectra further confirmed a significant relationship between conductivity and oxygen vacancy concentration. Lastly, depositing these In-doped SiC thin films onto p-type silicon substrates etched with buffered oxide etchant resulted in the formation of heterojunction p-n junction. This junction exhibited the rectifying characteristics of a diode, with sample current values in the vicinity of 10^2^ mA, breakdown voltage at approximately −5.23 V, and open-circuit voltage around 1.56 V. This underscores the potential of In-doped SiC thin films for various semiconductor devices.

## 1. Introduction

Ultrawide-bandgap (UWBG) semiconductor materials like silicon carbide (SiC) [1,2,3,4], gallium nitride (GaN) [5,6], and gallium oxide (Ga_2_O_3_) [7,8] are witnessing a resurgence marked by substantial progress in various domains. Recent research and development endeavors have unveiled profound insights into the properties and behaviors of UWBG materials. This deeper understanding has paved the way for breakthroughs in material synthesis, characterization, and manipulation, enabling researchers to harness their unique characteristics more effectively. The exploration of UWBG materials has extended beyond traditional applications, with researchers leveraging their exceptional properties to address emerging challenges in various fields. From high-power electronics to advanced optoelectronic devices, these materials are finding new and diverse applications, promising enhanced performance and efficiency in a wide range of technologies. Furthermore, the development of novel device concepts based on UWBG materials is driving innovation in semiconductor technology. These new devices, characterized by their UWBGs, offer unique capabilities and functionalities, opening up avenues for groundbreaking applications in areas such as power electronics [9,10], communications [10,11,12], and sensing [13]. The renaissance of UWBG semiconductor materials represents a significant paradigm shift in semiconductor research and technology. With ongoing advancements in material science, device engineering, and application development, these materials are poised to play a transformative role in shaping the future of electronics and beyond.

SiC semiconductors offer a multitude of advantages that make them highly desirable in various applications. First and foremost, SiC boasts an impressive melting point of over 2700 °C, more than double that of silicon, endowing it with exceptional high-temperature stability. This characteristic is particularly valuable in environments where extreme temperatures are encountered, ensuring reliable performance over extended periods. SiC semiconductors exhibit electron saturation drift velocities over ten times greater than silicon, translating to significantly higher operational frequencies. This attribute enables SiC-based devices to handle rapid switching and high-frequency operations with ease, making them ideal for advanced electronic systems requiring fast response times. In addition, SiC semiconductors possess superior breakdown field strengths compared to silicon, enabling them to withstand higher voltages. This capability leads to the realization of higher power densities, making SiC devices suitable for power electronics applications demanding robust performance under challenging conditions.

The inherent high-efficiency conversion properties and low power consumption of SiC further contribute to energy savings and carbon emission reduction. These characteristics align perfectly with the modern emphasis on green energy and environmental sustainability, making SiC a pivotal technology in advancing towards a cleaner and more efficient future. Furthermore, SiC semiconductors demonstrate stable performance even at elevated temperatures, making them well-suited for high-temperature applications like automotive power systems and power converters. This stability ensures reliable operation in demanding environments where traditional silicon-based devices may falter. Finally, the lower electron doping concentrations of SiC semiconductors result in reduced carrier concentrations, thereby minimizing circuit crosstalk and noise. This characteristic enhances the overall performance and reliability of SiC-based electronic systems, particularly in applications where signal integrity is critical. SiC semiconductors offer a comprehensive suite of advantages, ranging from high-temperature stability and high-speed operation to enhanced power handling capabilities and environmental sustainability. These qualities position SiC as a key enabler of next-generation electronic systems across a wide range of industries.

SiC semiconductors can achieve p-type and n-type conductivity by doping with different elements. Common dopants for p-type SiC semiconductors include aluminum [14] and boron [14,15], while nickel [2,16], phosphorus [17,18], and nitrogen [18] are frequently used dopants for n-type SiC semiconductors. Both p-type and n-type SiC semiconductors offer distinct advantages in various applications. P-type SiC semiconductors exhibit higher breakdown voltages and lower leakage currents, making them suitable for high-voltage and power electronic devices. On the other hand, n-type SiC semiconductors feature higher electron mobility and lower switching losses, making them ideal for high-speed and radio frequency (RF) electronic devices. The synthetic growth concept is an extensive approach addressing the growth behaviors of thin films and nanostructures. It has been employed successfully to elaborate the properties of inherently nanostructured carbon-based materials. This includes understanding the potentially varying morphology together with precursors and their role in the formation of nanostructured compounds and architectures in vapor-phase deposition techniques [19]. Also, Santos et al. offered valuable insights into the diverse atomic structures of indium oxide at the 2D level, highlighting variations in stoichiometry, notably 2D InO and 2D In_2_O_3_. Their research revealed that 2D InO displays a distinctive InSe-type structure, characterized by specific In–In distances [19,20]. This study aims to develop a straightforward method for depositing semiconducting silicon carbide (SiC) thin films. An innovative approach involves directly doping indium oxide (In_2_O_3_) into silicon carbide (SiC) powder (In_2_O_3_-doped SiC, abbreviated as In-doped SiC) to create 8.0 at% In-doped SiC powder. Subsequently, heating at 500 °C is employed to form a target, followed by utilizing electron beam (e-beam) technology to prepare In-doped silicon carbide thin films, a methodology scarcely explored in previous research.

Beyond these primary objectives, a significant focus of this study lies in characterizing the properties of In-doped silicon carbide films. Consequently, a comprehensive analysis was conducted, including X-ray diffraction (XRD), field-emission scanning electron microscopy (FEEM) with energy-dispersive spectroscopy (EDS) analysis, I-V characteristic analysis, transmittance analysis (applied to the Tauc Plot method for bandgap energy (Eg) value determination), X-ray photoelectron spectroscopy (XPS) analysis, and secondary ion mass spectrometry (SIMS) analysis. The results of the analysis reveal that although the In-doped silicon carbide films exhibit an amorphous phase, Hall effect measurements demonstrate excellent thin film resistivity, mobility, and carrier concentration, confirming their n-type semiconductor nature. Moreover, mapping analysis in electron microscopy EDS and SIMS analysis confirmed the uniform dispersion of In ions within SiC. Furthermore, the analysis indicated that the In_2_O_3_-doped silicon carbide films exhibited remarkable current density, with XPS analysis further substantiating a significant relationship between conductivity and oxygen vacancy concentration. The ultimate focus was on depositing these In_2_O_3_-doped silicon carbide films onto p-type silicon substrates to form heterojunction p-n junctions. These junctions exhibited diode-like rectifying characteristics, with sample current values in the vicinity of 10^2^, breakdown voltage at approximately −6V, and open-circuit voltage around 1.9 V. This underscores the potential applicability of In_2_O_3_-doped SiC thin films in semiconductor device technology.

## 2. Deposition of n-Type SiC Thin Films and Fabrication of Heterojunction n-Type SiC-p-Type Si Diodes

**Figure 1:**
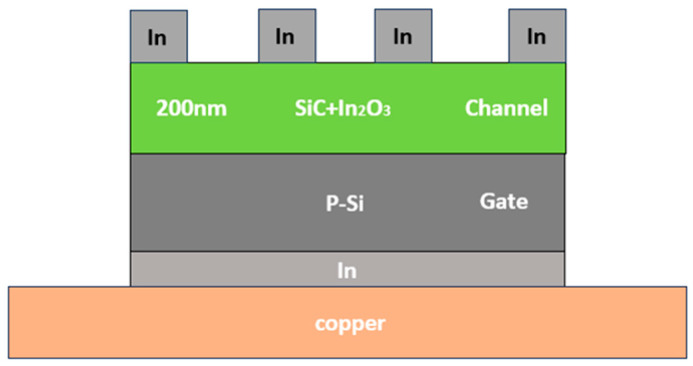
The investigated In-doped SiC thin films and manufacture of heterojunction diodes.

To enhance the quality of the polished surfaces on single-sided polished sapphire substrates and p-type silicon substrates (100), initial cleaning involved removing any grease, contaminants, or organic residues using acetone. Subsequently, isopropanol was applied to eliminate any lingering acetone, followed by rinsing with deionized water to clear away remaining solvents. To prevent watermarks, the polished substrate surfaces were swiftly dried using a nitrogen gun. Following this, buffered oxide etchant (BOE) was utilized to etch the residual SiO_2_ layer on the silicon substrates. Next, for the preparation of 8 at% In-doped SiC, the appropriate amounts of SiC (Acme Electronics Coro., Taipei, Taiwan) and In_2_O_3_ (Nano Materials, Tucson, AZ, USA) powders were meticulously weighed and combined in a ball mill’s grinding bowl. A small quantity of water was introduced to facilitate cohesion, and thorough mixing ensued. After drying all the mixed powder, the resulting material was subjected to heating in a furnace at 500 °C for 1 h. The process commenced by placing the heated target into the e-beam’s crucible, followed by initiating vacuum evacuation. The deposition pressure was maintained at 7.8 × 10^−6^ torr, with a deposition rate set at 10 nm per minute, and the deposition process lasted approximately 20 min. Figure 1 presents a visual depiction of the two-dimensional profile of the device structure in this study.

FESEM was employed to offer detailed information regarding the surface and cross-sectional morphologies of the deposited In-doped SiC thin films with nanometer-scale resolution. EDS mapping, in conjunction with FESEM, facilitated the acquisition of elemental composition information, enabling the generation of spatial distribution maps of elements within the sample. This allowed for researchers to visualize variations in elemental composition. XRD was employed to analyze the crystal structure of the In-doped SiC thin films, providing insights into their crystalline characteristics. UV–Visible spectroscopy was employed to measure the transmission spectrum of the In-doped SiC thin films, a crucial step in determining the Eg value using methods such as the Tauc Plot. B1500A instrumentation was utilized to measure the current–voltage (I-V) characteristics of the In-doped SiC thin films, offering essential data on their electrical behavior. XPS analysis was employed to investigate the chemical state of the In-doped SiC thin films, providing valuable information on their surface chemistry. The SIMS technique played a vital role in detecting and quantifying the elemental and molecular composition of the In-doped SiC thin films, contributing to a comprehensive understanding of their chemical makeup. Lastly, Hall measurement was employed to determine the electrical properties of the semiconductors, focusing on parameters such as carrier concentration, mobility, and conductivity, crucial for assessing their performance in electronic devices.

## 3. Results

**Figure 2:**
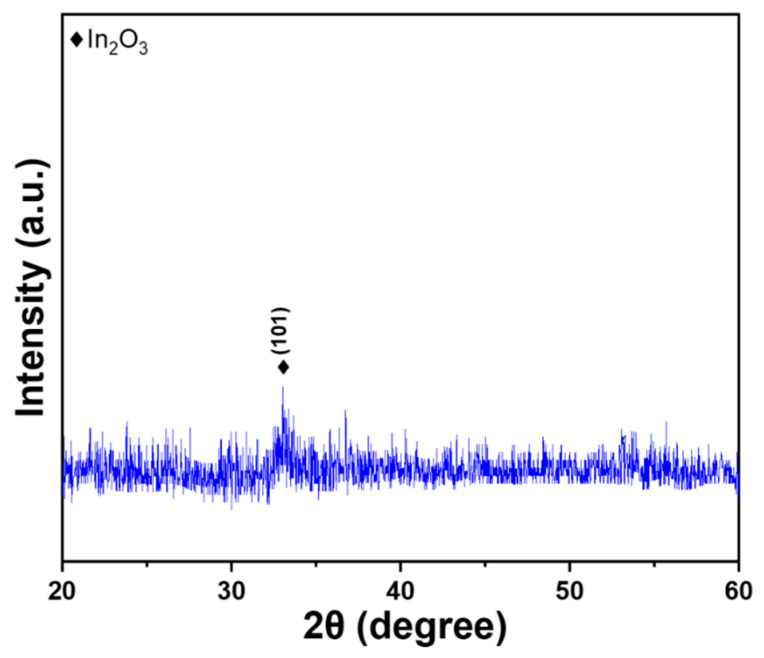
XRD pattern of the deposited In-doped SiC thin films.

The XRD spectrum provides a crucial insight into the structural characteristics of In-doped SiC thin films. In this study, XRD analysis was conducted on In-doped SiC thin films, revealing the crystalline phases present within the films. The results in Figure 2 indicate that the deposited In-doped SiC thin films only exhibit the (101) In_2_O_3_ phase. This finding suggests that the deposited SiC thin films may be amorphous, lacking a well-defined crystalline lattice. This could be attributed to low-temperature deposition without subsequent crystallization annealing. At room temperature, the In-doped SiC thin films only display the (101) In_2_O_3_ phase, primarily because the (101) crystal plane of In_2_O_3_ possesses lower surface energy [21]. Consequently, at room temperature, the (101) crystal plane of In_2_O_3_ is more stable and thus more likely to form. Moreover, the (101) crystal plane of In_2_O_3_ exhibits a higher lattice match with the (0001) crystal plane of SiC, facilitating the epitaxial growth of In_2_O_3_ films on SiC substrates. This outcome has implications for the conductivity of In-doped SiC thin films. Notably, the (101) crystal plane of In_2_O_3_ features higher carrier mobility and lower resistivity, making it an excellent candidate for transparent conductive thin film materials. Specifically, at room temperature, the lower surface energy of the (101) crystal plane of In_2_O_3_ suggests that In_2_O_3_ atoms tend to aggregate on this plane, resulting in lower energy for crystalline formation. Consequently, at room temperature, nucleation and growth of In_2_O_3_ are favored on the (101) crystal plane.

**Figure 3:**
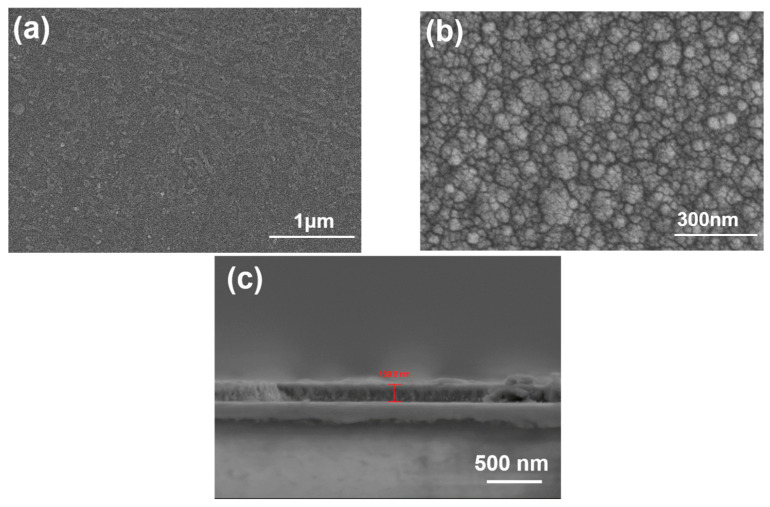
Surface observations of (**a**) SiC thin film deposited using e-beam, (**b**) In-doped SiC thin film deposited using e-beam, and (**c**) cross-sectional observation of In-doped SiC thin film.

Figure 3a presents surface observations of SiC thin films deposited using the e-beam, revealing a remarkably smooth surface. However, analysis of the I-V curve indicates that the SiC thin film is non-conductive, prompting the cessations of further analyses. In contrast, Figure 3b depicts surface observations of In-doped SiC thin films deposited using the e-beam, exhibiting the morphology of microgranules ranging in size from 1 to 100 nanometers. Furthermore, Figure 3c illustrates a cross-sectional view of the In-doped SiC thin film, providing insights into its thickness of approximately 189.8 nm. E-beam deposition is a technique that utilizes electron beam heating to evaporate materials and deposit them onto a substrate. E-beam deposition offers advantages such as uniform and dense coating, easy control of coating thickness, high purity of deposited materials, and suitability for various substrate materials. When using e-beam evaporation technology, the morphology and crystalline characteristics of thin films can be influenced by various factors. When depositing SiC thin films using the e-beam, a uniform and dense film of SiC material forms on the substrate. This is because SiC material has a higher evaporation temperature, and under e-beam heating, the SiC material evaporates fully and deposits evenly on the substrate.

Additionally, the flatness of the deposited SiC thin film may be due to the formation of a relatively uniform structure on the film surface. In_2_O_3_ + SiC thin film, on the other hand, is a composite material consisting of two materials: In_2_O_3_ and SiC. When depositing In_2_O_3_ + SiC thin films using the e-beam, the evaporation temperatures of In_2_O_3_ and SiC materials differ. The In_2_O_3_ material has a lower evaporation temperature, so under e-beam heating, the In_2_O_3_ material preferentially evaporates. On the other hand, the SiC material has a higher evaporation temperature, so under e-beam heating, the SiC material begins to evaporate only after the In_2_O_3_ material has evaporated. In the In_2_O_3_ + SiC system, there may exist different types of nuclei, resulting in an uneven structure on the film surface with the appearance of microgranules. This is because when the In_2_O_3_ material deposits on the substrate, the SiC material has not yet evaporated; thus, the In_2_O_3_ material forms nuclei on the substrate and continues to grow on them. After the SiC material evaporates, it deposits onto the In_2_O_3_ nuclei, and the chemical interaction between In_2_O_3_ and SiC results in the formation of an uneven structure on the film surface, leading to microgranules.

**Figure 4:**
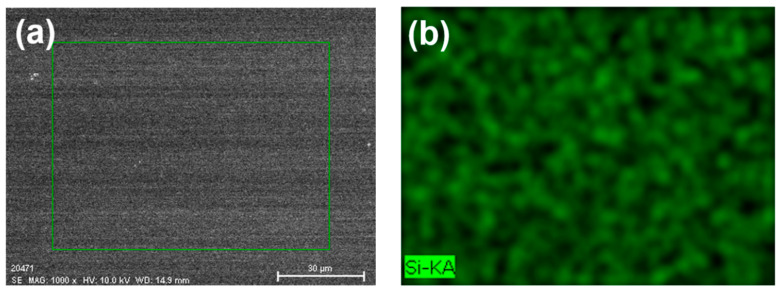

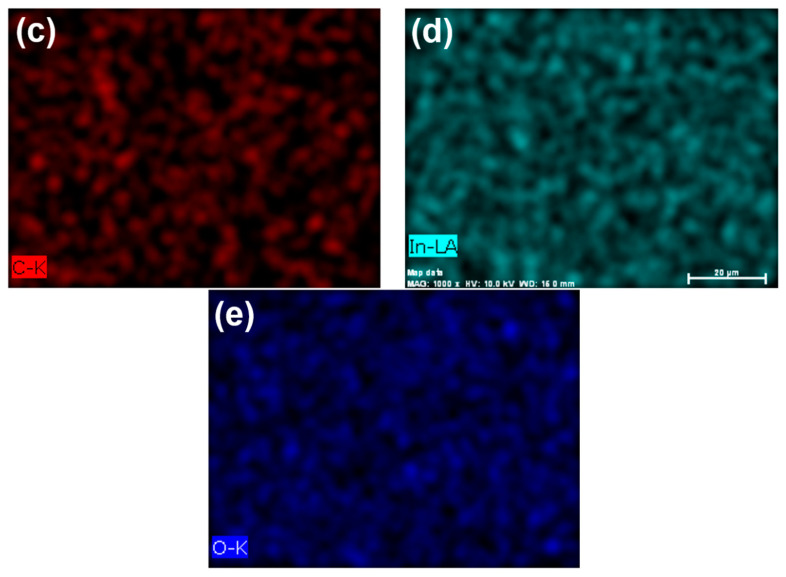
(**a**) An image of the surface area of an In-doped SiC thin film under analysis and (**b**–**e**) the distributions of Si, C, In, and O elements on the analysis area.

EDS mapping stands as a formidable tool for probing the elemental composition of materials at a microscopic scale. Its versatility extends to the examination of diverse phenomena, including elemental distribution within materials, inter-material element diffusion, and the genesis of novel phases. In our study, EDS mapping was employed to ascertain the uniformity of an In-doped SiC thin film deposited through e-beam technology using In_2_O_3_ + SiC as the precursor material. Figure 4a presents an image of the surface under analysis, with elements such as Si, C, In, and O subjected to scrutiny, as illustrated in Figure 4b–e. This analytical approach facilitated a detailed observation of the elemental distribution across various regions of the thin film’s surface, thereby further corroborating its uniformity. The results depicted in Figure 4b–e reveal a homogeneous distribution of elements, including Si, C, In, and O, across the analyzed surface. This outcome not only attests to the uniformity of the In-doped SiC thin film, but also underscores the efficacy of utilizing In_2_O_3_ + SiC as precursor materials and e-beam technology for depositing high-quality, homogeneous thin films. In essence, the application of EDS mapping in this study not only validates the uniform distribution of elements within the In-doped SiC thin film, but also highlights the potential of the employed deposition method and precursor materials for achieving desired material properties crucial for various applications.

**Figure 5:**
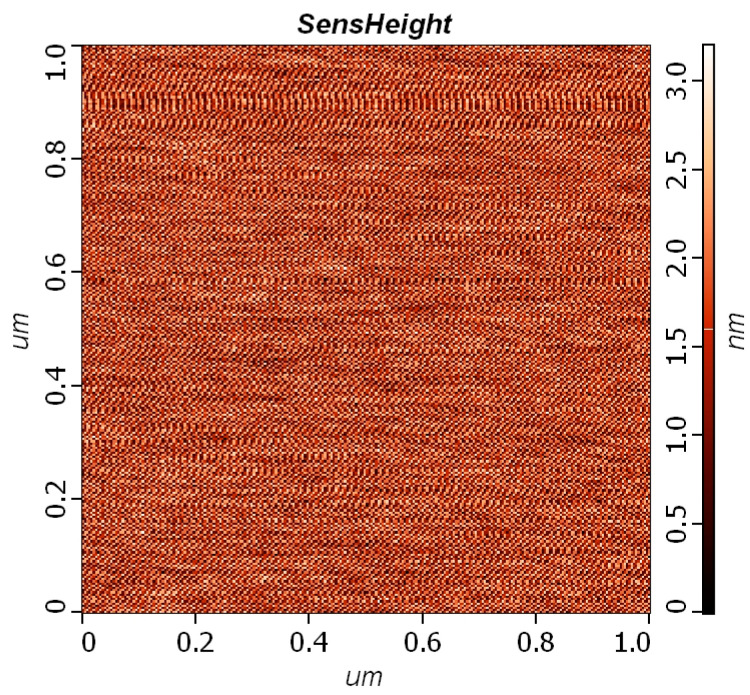
AFM analysis of the deposited In-doped thin film.

To assess the efficacy of the evaporation method in producing high-performance semiconductive devices, we utilized AFM to examine the surface characteristics of the deposited In-doped thin film. The findings are illustrated in Figure 5. The measured Sq, Sa, Ra, and Rq values were determined to be 0.7321, 0.6338, 0.6546 nm, and 1.7854 nm, respectively. Sq, or the root-mean-square roughness, offers insights into the overall fluctuation or undulation of surface height deviations. Sa, representing the surface arithmetic mean, provides a measure of the average deviation of surface heights across the entire scanned area. Ra, which denotes the average roughness of the surface, indicates the average deviation of surface heights within a specified measuring length. The Rq value represents the root-mean-square roughness; it is a commonly used parameter to characterize surface roughness. These parameters collectively contribute to our understanding of the surface quality and topographical features of the deposited thin film, essential for evaluating its suitability for semiconductive device fabrication.

**Figure 6:**
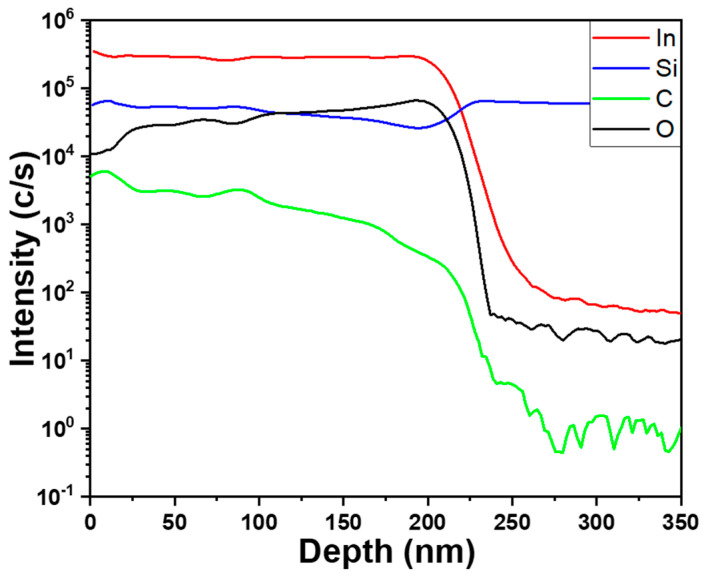
SIMS analysis results of Si, C, In, and O elements of an In-doped SiC thin film.

Figure 6 illustrates the SIMS analysis results of elements such as Si, C, In, and O. From the SIMS analysis, it can be observed that the signals (concentration) of Si and C gradually decrease from the surface. At a depth of approximately 195 nm, the concentration of C starts to decrease rapidly, while that of Si begins to slightly increase. The increase in Si concentration is attributed to the analysis depth reaching the Si substrate. Conversely, the concentration of In shows no significant variation from the surface to a depth of 195 nm. As for O, its concentration slowly increases from the surface of the thin film, but notably begins to decrease rapidly around 195 nm in depth. First, these analysis results validate the accuracy of the SEM film thickness image. These results match the thickness of the deposited In-doped SiC thin films: approximately 189.8 nm. Furthermore, the gradual decreases in Si and C concentrations indirectly support the earlier explanation that In_2_O_3_ components evaporate first during deposition, while SiC evaporates more slowly. Hence, the concentrations of Si and C increase from the bottom of the film. Additionally, the gradual increase in O components from the surface inward indicates that during the deposition of In_2_O_3_, partial decomposition into In and O occurs, with the latter evaporating to form oxygen vacancies. This aspect may influence the conductivity of the In-doped SiC thin films. In this study, the transformation of completely non-conductive SiC into conductive In-doped SiC thin films further underscores the importance of our research.

Past research has highlighted the persistent challenge of achieving uniform dispersion of indium (In) ions in the realm of SiC materials. One of the contributing factors to this difficulty lies in the disparate physicochemical properties between indium atoms and silicon (Si) and carbon (C) atoms. This may be attributed to the lower electronegativity of In atoms (1.7) compared to Si (2.5) and C (1.8) atoms, as well as the lower electron affinity of In atoms (−0.34 eV) compared to Si (−1.34 eV) and C (−1.22 eV) atoms. These differences could potentially induce repulsive effects of In ions within the SiC lattice, thereby impeding uniform distribution. However, this study successfully achieves the uniform dispersion of In ions in SiC, marking a significant advancement. The primary reason for this achievement lies in the utilization of In_2_O_3_ as a dopant in this research, resulting in the dispersion of In_2_O_3_ within SiC. The formation of In ions occurs during the e-beam deposition process, wherein a portion of In_2_O_3_ decomposes. This finding not only contributes to the understanding of material properties, but also holds promise for applications in electronic devices.

**Figure 7:**
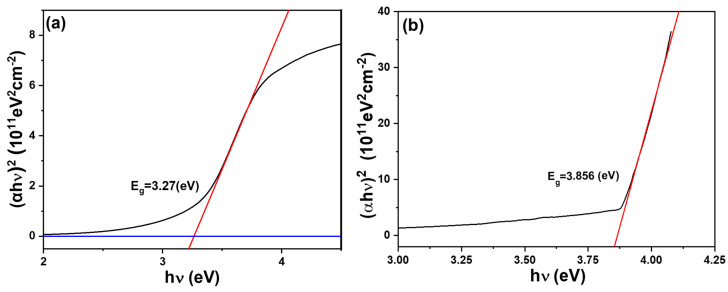
Tauc Plot of (**a**) a pure SiC thin film and (**b**) an In-doped SiC thin film.

Transmittance, often employed to characterize a material’s ability to allow specific wavelengths of light to pass through, plays a crucial role in understanding optical properties. In solid materials, particularly semiconductors, transmittance correlates with the bandgap—a fundamental parameter dictating a material’s behavior in light absorption and transmission. This study focuses on measuring the relationship between transmittance and wavelength and subsequently employs the Tauc Plot method to determine the Eg values of pure SiC and In-doped SiC thin films. The Tauc Plot method stands as a widely adopted technique, especially effective for semiconductor and insulator materials. It relies on transmission spectra, plotting the relationship between the optical absorption coefficient (α) and the photon energy (hν) across various wavelengths of light. In this graphical representation, the bandgap corresponds to the intercept of the linear portion between α squared and hν. According to the Tauc Plot method, we determine that the deposited pure SiC and 8.0 at% In-doped SiC thin films exhibit the approximate bandgaps of 3.27 eV and 3.856 eV, as Figure 7a,b show. The Eg value of SiC thin film is about 3.3 eV [22] and the Eg value of In_2_O_3_ thin film is about 3.5 eV [23].

The higher bandgap of In-doped SiC thin films compared to that of In_2_O_3_ and SiC thin films may be attributed to the differences in the crystal structure and composition of the materials. In the context of SiC, different crystal structures, such as the 4H-SiC structure, offer advantages like higher bandgaps and faster electron mobility, making them suitable for power device manufacturing. On the other hand, the doping of materials like In_2_O_3_ thin film can affect their properties, potentially altering their bandgap and conductivity characteristics. Therefore, the higher bandgap of In-doped SiC thin films could be a result of the specific doping process and the unique combination of properties from both In_2_O_3_ and SiC thin films, leading to enhanced bandgap characteristics suitable for various applications requiring high efficiency and performance in power electronics and semiconductor devices. This research underscores the importance of understanding the optical properties of materials. Particularly in the realm of semiconductor technology, precise control over bandgap characteristics is paramount for numerous applications. These applications span from optoelectronics to photovoltaics.

**Figure 8:**
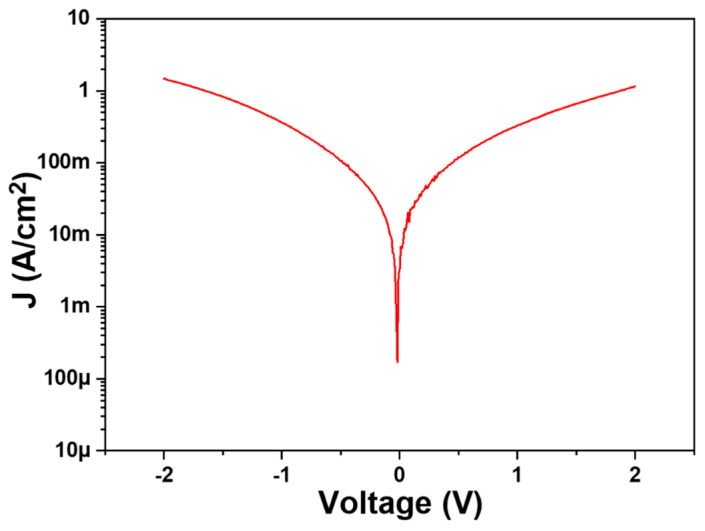
Current–voltage property of an In-doped SiC thin film.

This study aimed to enhance the conductivity of SiC thin films, which inherently lack conductivity, by doping them with In_2_O_3_. The electrical performance of In-doped SiC films was evaluated using B1500A electrical analysis. Experimental results revealed that within the measurement range of −2V to +2V, the In-doped SiC thin films with the thickness of approximately 189.8 nm exhibited a certain level of conductivity. Specifically, when 8 at% In was used as the doping concentration for SiC, when the bias voltage was −2V or +2V, the current density reached the order of 10^0^/cm^2^, as Figure 8 shows. Several factors may contribute to the formation of n-type semiconductor behavior in In_2_O_3_-doped SiC. First, In_2_O_3_ is known as an n-type semiconductor material, with its conductivity attributed to oxygen vacancies. These vacancies capture electrons, generating free electrons and thus rendering In_2_O_3_ n-type conductive. This aligns well with the results obtained from SIMS analysis. Second, SiC is a wide-bandgap semiconductor material with a low intrinsic carrier concentration. When In_2_O_3_ is doped into SiC, the oxygen vacancies in In_2_O_3_ provide free electrons to SiC, thereby enhancing its n-type conductivity. Lastly, the mismatch in lattice constants between In_2_O_3_ (approximately 10.1 Å) and SiC (approximately 4.3 Å) results in lattice distortion upon In_2_O_3_ doping into SiC, leading to interface defects. These defects also contribute free electrons to SiC, thereby enhancing its n-type conductivity. Understanding the mechanisms behind the enhanced conductivity of In-doped SiC is crucial for the development of novel semiconductor materials. Further investigation could explore the electrical performance of SiC films with different doping concentrations of In_2_O_3_ and evaluate their feasibility and efficiency in practical applications.

The measurement of current–voltage property revealed that the electrical characteristics of the SiC thin films doped with In_2_O_3_ were exceptionally good. To obtain more data, Hall measurements were conducted on the thin films. The measurement results revealed that the carrier concentration of SiC thin films doped with 8 at% In reached 5.20 × 10^19^/cm^3^, with a resistivity of 2.04 × 10^−4^ and a mobility of 287 cm^2^/Vs. By calculating the electrical conductivity of the thin films based on these values, it was confirmed that SiC thin films doped with 8 at% In indeed exhibit excellent conductivity. The Hall parameters (carrier concentration and mobility) of SiC thin films and In_2_O_3_ are influenced by various factors such as material purity, doping concentration, film thickness, and fabrication methods. These factors play a significant role in determining the electrical properties of the films and highlight the complexity involved in optimizing their conductivity for practical applications. Continued investigation into these factors will contribute to a deeper understanding of the behavior of In-doped SiC thin films and facilitate their advancement in electronic and semiconductor technologies.

Previous research has indicated that the carrier concentration of SiC thin films typically ranges from 10^14^/cm^3^ to 10^18^/cm^3^. The carrier concentration of n-type SiC thin films is primarily determined by the dopant atomic concentration, such as nitrogen, while that of p-type SiC thin films is mainly influenced by the atomic concentration of the dopant such as phosphorus. The electron mobility of SiC thin films typically falls between 100 cm^2^/Vs and 1000 cm^2^/Vs, whereas the hole mobility typically ranges from 50 cm^2^/Vs to 500 cm^2^/Vs. Both electron and hole mobilities are subject to the influence of SiC thin film crystal quality and defect density. In_2_O_3_ typically exhibits a carrier concentration ranging from 10^18^/cm^3^ to 10^21^/cm^3^, and the carrier concentration of In_2_O_3_ is primarily determined by oxygen vacancies. The electron mobility of In_2_O_3_ generally ranges from 30 cm^2^/Vs to 100 cm^2^/Vs, and the electron mobility is affected by the crystal quality and defect density of In_2_O_3_. Understanding these parameters for both SiC and In_2_O_3_ is pivotal for assessing their applicability in various fields, particularly in semiconductor device fabrication. These parameters of the In-doped SiC thin films provide crucial insights into their electrical behavior, aiding in their optimization for enhanced performance.

Furthermore, investigating the impacts of crystal quality and defect density on carrier mobility can contribute to improving the reliability and efficiency of electronic devices incorporating these materials. Therefore, this study further utilized XPS to investigate the chemical composition and electronic structure of the In-doped SiC thin films. This involved utilizing the photon energy and intensity data obtained from XPS to gather information about the material’s surface chemical composition and electronic states, including the types of elements present, the states of chemical bonds, and the distribution of electronic energy levels. XPS is a powerful quantitative technique and it is particularly valuable for discerning not only which elements are present within deposited films but also the specific chemical bonds they form with other elements. In the context of investigating the electrical properties of In-doped SiC thin films, XPS was employed to precisely characterize the chemical bonding states of carbon (C), oxygen (O), silicon (Si), and indium (In) elements. By leveraging these measured results, researchers sought to elucidate the underlying factors contributing to variations in the electrical behavior of the films.

The peaks of the C_1s_ element can be divided into the following three main components:

(1) Carbon–Carbon (C-C or C_I_) peak: Typically located around 284.5 eV, it represents covalent bonds formed between carbon atoms.
(2) Carbon–Oxygen (C-O or C_II_) peak: Located within the range of 287–289 eV, it represents chemical bonds formed between carbon and oxygen atoms.
(3) Carbon–Silicon (C-Si or C_III_) peak: If there are chemical bonds between carbon and silicon in the sample, this peak may occur at higher energy levels, usually between 290 and 294 eV.

**Figure 9:**
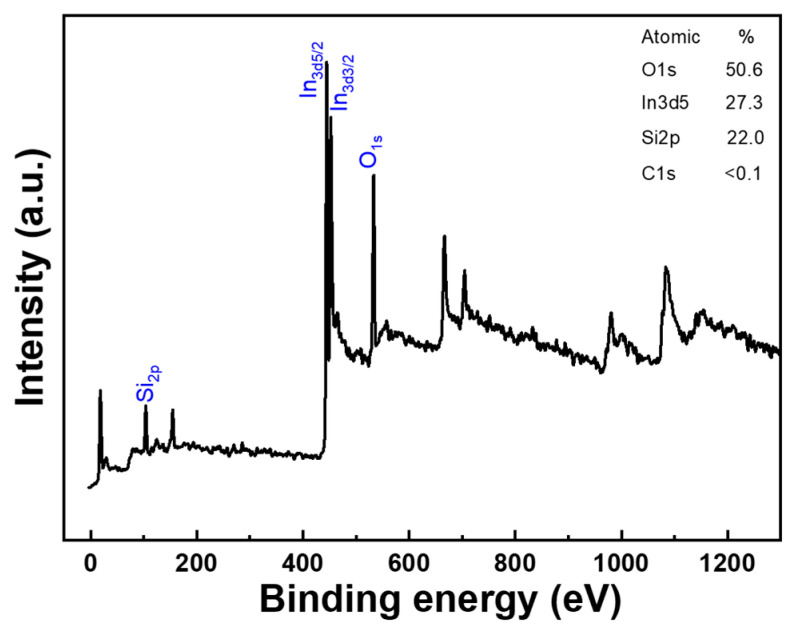
XPS analysis spectrum of the In-doped SiC thin films.

Figure 9 shows the XPS analysis spectrum of the In-doped SiC thin films, but conspicuously, the C_1s_ element peak is absent in the XPS analysis. The stability of the SiC compound could be one of the reasons for the absence of the C_1s_ element peak in XPS analysis. For instance, on the surface of the diamond, the bonding energy of C-C bonds is high, hence the C_1s_ peak is not observed in the XPS spectrum. SiC is an extremely stable compound, with bonds between carbon and silicon typically being covalent, imparting high chemical stability. In XPS analysis, the C_1s_ signal usually arises from carbon compounds on the sample surface, but due to the stability of SiC, its C_1s_ signal may not be easily detected. The stability of SiC might hinder the breaking of chemical bonds between carbon and silicon atoms in XPS analysis, thus the C_1s_ signal may not appear or be very weak. In such cases, even if carbon elements are present in SiC, the corresponding C_1s_ peak may not be observable through XPS. Therefore, the stability of the SiC compound presents a potential reason for the absence of the C_1s_ element peak in XPS analysis.

**Figure 10:**
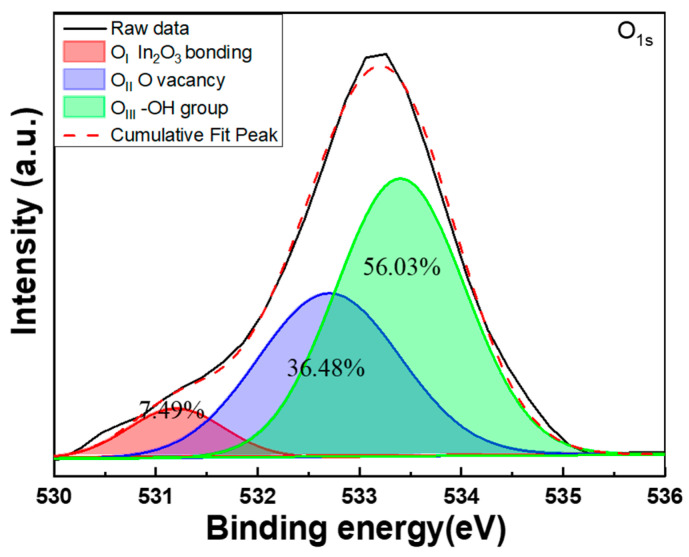
XPS spectrum and Gaussian-resolved spectra of the O_1s_ peak for In-doped SiC thin films.

Figure 10 displays the XPS spectrum of the O_1s_ peak, centered at 533.3 eV, along with the Gaussian-resolved spectra of the O_1s_ peak for the In-doped SiC thin films. The typical surface O_1s_ peak of the In-doped SiC thin films can be modeled by three distinct Gaussian components: O_I_, O_II_, and O_III_ peaks. These three peaks of the O_1s_ signal are centered at 530.6 eV (O_I_), 531.2 eV (O_II_), and 533.4 eV (O_III_), respectively. In the XPS analysis of the In-doped SiC thin films, the observed peaks—O_I_, O_II_, and O_III_—each signify distinct oxidation states of oxygen within the material. The O_I_ peak corresponds to oxygen fully bonded to silicon atoms, forming silicon dioxide (SiO_2_) [24]. This oxide is insulating in nature, meaning it does not contribute to the conductivity of the SiC thin film. It serves as a stable and inert component within the material. Meanwhile, the O_II_ peak indicates oxygen that is partially bonded to silicon, forming suboxides such as SiO or Si_2_O, which can cause oxygen vacancy within the In-doped SiC thin films [25]. These suboxides can exhibit varied behavior ranging from insulating to conducting depending on their stoichiometry and structural arrangement. Importantly, suboxides have the potential to introduce charge carriers into the SiC lattice, thereby influencing its conductivity. However, the impact of suboxides on conductivity is multifaceted and hinges on several factors, including the specific composition and structure of the suboxide species and the doping concentration present in the SiC thin film.

Lastly, the O_III_ peak corresponds to oxygen not bonded to silicon atoms but instead adsorbed onto the surface of the SiC thin film [26]. Unlike the other peaks, adsorbed oxygen does not directly influence the conductivity of the material; it merely resides on the surface without altering its electrical properties. Of the three peaks observed, the O_II_ peak stands out as the most likely contributor to changes in the conductivity of the SiC thin film. Its potential to introduce charge carriers into the lattice underscores its significance in understanding and controlling the electrical behavior of the material. However, the intricate interplay of factors, including the nature of suboxides and the doping characteristics, necessitates careful analysis to fully comprehend their impact on conductivity. The presence of a prominent O_II_ peak in an XPS spectrum does not automatically denote material conductivity. Instead, it may suggest the existence of oxygen vacancies, which could potentially enhance conductivity. Thus, observing an O_II_ peak at 531.2 eV in the XPS spectrum provides compelling evidence supporting the material’s conductive properties, as it signifies the probable presence of these vacancies.

**Figure 11:**
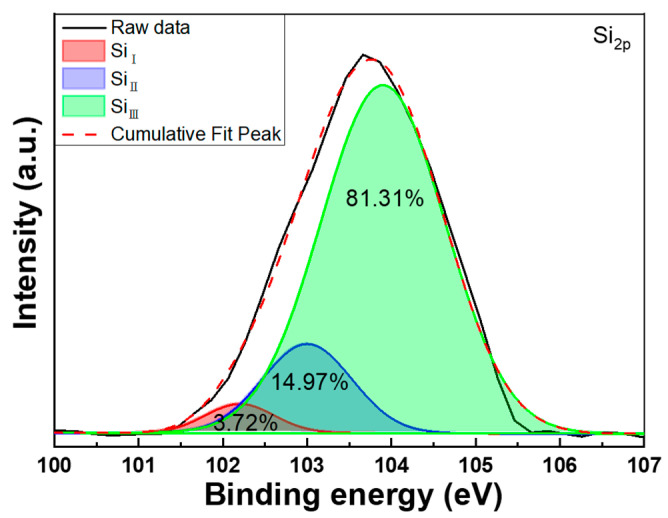
XPS spectrum and Gaussian-resolved spectra of the Si_2p_ peak for In-doped SiC thin films.

In the XPS analysis of the In-doped SiC thin films, the Si_2p_ XPS spectra typically display three prominent peaks, designated as Si_I_, Si_II_, and Si_III_, as illustrated in Figure 11. These peaks signify distinct chemical environments of silicon atoms within the material. The Si_I_ peak, situated approximately at 102.2 eV, signifies silicon atoms bonded to two silicon atoms and two oxygen atoms. This peak is indicative of deeper oxide layers or SiO_2_ compounds present in the material. The Si_II_ peak, positioned around 103.0 eV, is associated with silicon atoms bonded to three silicon atoms and one oxygen atom. This peak suggests the existence of surface oxide layers or SiO_x_C compounds on the SiC surface. Lastly, the Si_III_ peak, located at approximately 103.9 eV, corresponds to silicon atoms bonded to four other silicon atoms, constituting the SiC network. This particular species is the most prevalent within SiC thin films. In XPS analysis of In-doped SiC thin films, the SiII peak serves as a vital indicator for assessing changes in the material’s conductivity. This peak specifically corresponds to silicon in an oxidized state, predominantly found in compounds like SiO_2_. The formation of silicon oxide can act as an insulating layer, thereby diminishing the overall conductivity of the SiC thin film.

In these thin films, SiC inherently exhibits semiconductor properties due to its unique band structure, allowing for the flow of electrical current facilitated by free electrons in the conduction band. However, when subjected to oxidation, as is the case with the In-doped SiC thin films, silicon oxide (SiO_2_) forms on the surface. SiO_2_ lacks free electrons, rendering it an insulator that impedes the flow of electrical current. The presence and intensity of the Si_II_ peak in XPS spectra directly correlate with the extent of silicon oxide formation. As the Si_II_ peak intensity increases, indicative of greater SiO_2_ formation, the conductivity of the SiC thin film decreases. Consequently, by monitoring the Si_II_ peak intensity in XPS analysis, one can indirectly evaluate alterations in the conductivity of the In-doped SiC thin films. A heightened SiII peak intensity suggests reduced conductivity due to an augmented presence of insulating SiO_2_. However, it is essential to note that conductivity changes in the In-doped SiC thin films can be influenced by factors beyond SiO_2_ formation. Parameters such as the doping concentration and distribution of indium atoms also play significant roles in determining conductivity variations. Thus, while the Si_II_ peak offers valuable insights into conductivity alterations associated with silicon oxide formation, comprehensive analysis incorporating other influencing factors is imperative.

**Figure 12:**
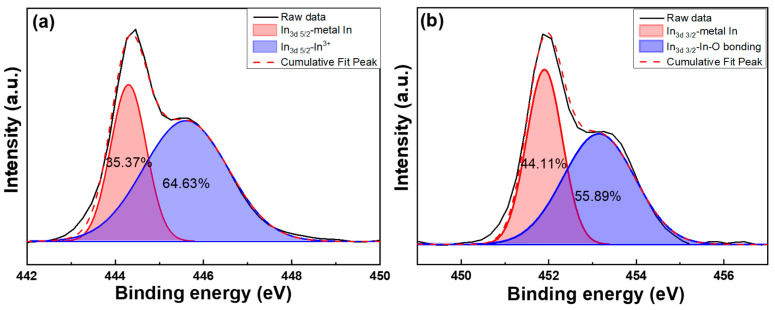
XPS spectra and Gaussian-resolved spectra of (**a**) In_3d5/2_ and (**b**) In_3d3/2_ peaks for the In-doped SiC thin films.

Figure 12a,b display the typical surface XPS spectra and Gaussian-resolved spectra of the In_3d5/2_ and In_3d3/2_ peaks for In-doped SiC thin films, respectively. The binding energies of the In_3d5/2_ and In_3d3/2_ peaks are found at 444.5 eV and 451.9 eV, respectively. Previous research has extensively examined the binding energies of electron states In_3d5/2_ and In_3d3/2_ associated with both indium oxide (In_2_O_3_) and metallic indium. For indium oxide, these energies have been determined as 444.0 eV and 451.6 eV, respectively [27], while for metallic indium, the corresponding values are slightly lower, recorded at 443.7 eV and 451.1 eV [28]. This comparison sheds light on the subtle variations in electron binding energies between the oxide and metallic forms of indium, suggesting potential implications for their electronic structure and chemical behavior. Notably, there is no significant difference in the binding energies of the In_3d5/2_ and In_3d3/2_ peaks compared to previous studies. The most significant deviation from past analyses is evident in the XPS spectra of the In-doped SiC thin films, where the In_3d5/2_ and In_3d3/2_ peaks are distinctly split into two sub-peaks [29,30]. Distinct results were observed in the XPS analysis of the In_2_O_3_-SiC thin film, where two subpeaks of the In_3d5/2_ and In_3d3/2_ peaks are prominently displayed. This anomaly could be attributed to various factors. One possible explanation could be related to the specific composition or structure of the In_2_O_3_-SiC thin film, leading to unique chemical bonding environments for the indium atoms. This could result in the splitting of the In_3d5/2_ and In_3d3/2_ peaks into two distinct subpeaks, indicating different chemical states or coordination environments of the indium atoms within the film. Additionally, the interaction between indium oxide In_2_O_3_ and SiC in the thin film could also play a significant role in influencing the XPS results.

The presence of silicon carbide may introduce additional complexities in the electronic structure of the film, affecting the XPS spectra and leading to the observed distinct subpeaks in the In_3d5/2_ and In_3d3/2_ peaks. This nuanced distinction sheds light on the intricate doping effects influencing the material’s characteristics, providing valuable insights for further investigation and application development. In this study, the In_3d5/2_ peak was notably resolved into two distinct peaks at 451.9 eV and 453.1 eV, while the In_3d3/2_ peak exhibited a bifurcation into two peaks at 444.3 eV and 445.8 eV, respectively. This clear decomposition of the peaks indicates the presence of distinct electronic states or chemical environments for the indium species under investigation. In previous research, Wang et al. found that the first peak was associated with the indium peak characteristic of the ITO structure (In-O, structural O), while the second peak was attributed to the presence of indium associated with the indium hydroxide structure (In-OH, structural OH) [30]. However, our research focuses on a system where the primary materials consist of In_2_O_3_ incorporated into SiC. Consequently, the probability of OH bonds being present is minimal. For the In_3d5/2_ and In_3d3/2_ peak, the first peaks at 451.9 eV and 444.3 eV indicate the presence of the In^3+^ oxidation state at the interface of In_2_O_3_-SiC, and the second peaks at 453.1 eV and 445.6 eV correspond to the In^3+^ oxidation state within the In_2_O_3_ structure itself.

Table 1 provides a detailed comparison of XPS analysis results for the O_1s_, Si_2p3/2_, and In_3d_ electronic states in the In_2_O_3_-SiC thin film. These data offer a comprehensive understanding of the electronic structure and chemical environment of different species in the samples. At the O_1s_ level, oxide samples exhibit characteristic peak energies and shapes, reflecting the chemical states of oxygen atoms and their bonding environments. Similarly, the Si_2p3/2_ level offers crucial information about the chemical states of silicon atoms in silicon compounds and their bonding modes with other elements. Meanwhile, the In_3d_ level provides insights into the differences in indium’s electronic structure among various samples, revealing subtle distinctions between its oxide and metallic states. This comparative analysis of XPS data deepens our understanding of the chemical property differences among oxide samples and related materials, serving as a valuable reference for further exploration of their applications in fields such as electronic devices and catalysts.

**Figure 13:**
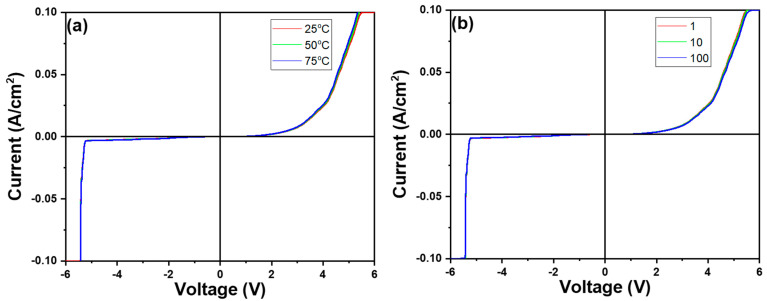
I-V characteristic measurement of the investigated heterojunction diode under (**a**) different temperatures and (**b**) different times.

After conducting a comprehensive analysis of the physical and electrical properties of In-doped SiC thin films, we proceeded to measure their performance using a specialized device structure. The device structure of the heterojunction diode depicted in Figure 1 was employed for conducting I-V characteristic measurement. The results of these measurements, as presented in Figure 13, clearly exhibit diode-like characteristics. The forward voltage drop (defined at current density = 1 mA/cm^2^) of the device is 1.56 V, while the reverse breakdown voltage is −5.23 V. It is evident that the In-doped SiC thin film exhibits n-type behavior, thus forming a heterojunction diode with the p-type Si substrate. During testing, the outstanding electrical performance and thermal stability of the In-doped SiC thin film became apparent, suggesting potential advantages when applied in heterojunction diodes. These advantages may include lower reverse leakage current under reverse bias and higher power handling capabilities. Therefore, through detailed analysis and measurement of the device characteristics, we can gain a better understanding of its potential performance in various applications, providing insights for future research and applications. This particular heterojunction diode structure holds promise for applications in high-power, high-frequency, and high-temperature environments. For instance, in high-power electronic devices, this heterojunction diode can offer reduced switching losses and higher operating frequencies, thereby enhancing the efficiency and performance of the entire system.

The I-V characteristic measurements under different temperatures (25 °C, 50 °C, 75 °C) or different numbers of measurements at room temperature (1 time, 10 times, 100 times) are also shown in Figure 13a,b. Clearly, as shown in Figure 13a, with increasing temperature, the forward voltage slightly decreases while the breakdown voltage remains almost unchanged. Similarly, as illustrated in Figure 13b, with an increase in the number of measurements, the forward voltage slightly increases while the breakdown voltage also remains almost unchanged. These observations suggest that temperature has a modest effect on the forward voltage, indicating a slight decrease with increasing temperature, while the breakdown voltage appears to be relatively insensitive to temperature changes. Additionally, the number of measurements does not significantly alter the breakdown voltage, although there is a slight increase in the forward voltage with an increasing number of measurements, possibly due to the conditioning or slight changes in the measurement setup over multiple runs. Additionally, the thermal stability of In-doped SiC thin films in the higher temperature environments ensures the stability and reliability of the device, expanding its application range. The results also indicate that devices fabricated from In-doped SiC thin films exhibit high longevity. This observation suggests that the incorporation of indium doping into the silicon carbide thin films contributes to the robustness and reliability of the devices.

## 4. Conclusions

The XRD pattern revealed that the deposited In-doped SiC thin films, which had the thickness of approximately 189.8 nm, exclusively displayed the (101) phase of In_2_O_3_. Surface observations further indicated that the deposited SiC thin films exhibited a notably smooth surface, while the In-doped SiC thin films showcased a granular crystalline morphology. These findings substantiate that the inclusion of In_2_O_3_ alters the properties of the deposited SiC thin films. According to the Tauc Plot method, the deposited In-doped SiC thin film demonstrated an estimated bandgap of approximately 3.89 eV. Additionally, Hall measurements showed that the carrier concentration of the SiC thin films doped with 8 at% In reached 5.20 × 10^19^/cm^3^, accompanied by a resistivity of 2.04 × 10^−4^ Ω·cm and a mobility of 287 cm^2^/Vs. SIMS analyses revealed a gradual decrease in the concentrations of Si and C from the surface. Around a depth of approximately 195 nm, C concentration experienced a rapid decline, while Si concentration showed a slight increase. This increase in Si concentration is attributed to the analysis depth reaching the Si substrate. Conversely, the concentration of In showed no significant variation from the surface to a depth of 195 nm. Regarding O, its concentration slowly increased from the surface of the In-doped SiC thin films but notably began to decrease rapidly around 195 nm in depth. In the XPS spectrum, the C_1s_ peak was not observed, while the Si_2p_ XPS spectra typically displayed three prominent peaks designated as Si_I_, Si_II_, and Si_III_, with binding energies of 102.2 eV, 103.0 eV, and 103.9 eV. The O_1s_ peak was further characterized by three distinct Gaussian components: O_I_, O_II_, and O_III_ peaks, with binding energies of 530.6 eV, 531.2 eV, and 533.4 eV, respectively. As for the heterojunction diode, its forward voltage drop, defined at a current density of 1 mA/cm^2^, was measured at 1.56 V, while the reverse breakdown voltage was determined to be −5.23 V.

## Figures and Tables

**Table 1:** The detailed comparison of XPS analysis results for the O_1s_, Si_2p3/2_, and In_3d_ electronic states in the In_2_O_3_-SiC thin film.

O_1s_	O_I_ (In-O bonding)				O_II_ (O vacancy)				O_III_ (absorption O on surface)			
	530.6 eV/7.49%				531.2 eV/36.48%				533.4 eV/56.03%			
Si_2p3/2_	Si_I_ (Si-C bonding)				Si_II_ (Si-O bonding)				Si_III_ (Si-O_2_ bonding)			
	102.2 eV/3.72%				103.0 eV/14.97%				103.9 eV/81.31%			
In_3d_	In_3d3/2_ (metal In)			In_3d3/2_ (In_2_O_3_)			In_3d5/2_ (metal In)			In_3d5/2_ (In_2_O_3_)		
	451.9 eV/44.11%			453.1 eV/55.89%			444.3 eV/35.37%			445.8 eV/64.63%		

## Data Availability

No new data were created or analyzed in this study. Data sharing is not applicable to this article.
